# Machine learning and AVO class II workflow for hydrocarbon prospectivity in the Messinian offshore Nile Delta Egypt

**DOI:** 10.1038/s41598-025-86765-7

**Published:** 2025-01-28

**Authors:** Nadia Abd-Elfattah, Aia Dahroug, Manal El Kammar, Ramy Fahmy

**Affiliations:** 1 Geophysics Department, Cairo University, Cairo, Egypt; 2 Rashpetco Company, Cairo, Egypt

**Keywords:** Machine learning, Seismic, AVO, Miocene, Nile Delta, Gas sand, Geophysics, Stratigraphy

## Abstract

This study presents a comprehensive workflow to detect low seismic amplitude gas fields in hydrocarbon exploration projects, focusing on the West Delta Deep Marine (WDDM) concession, offshore Egypt. The workflow integrates seismic spectral decomposition and machine learning algorithms to identify subtle anomalies, including low seismic amplitude gas sand and background amplitude water sand. Spectral decomposition helps delineate the fairway boundaries and structural features, while Amplitude Versus Offset (AVO) analysis is used to validate gas sand anomalies. The entire seismic volume is classified into facies domains using machine learning, which isolates target features from seismic background data. The study area, covering 1850 km^2^, includes major structures such as the Rosetta fault and Nile Delta offshore anticline, with reservoirs consisting of layered sandstones and mudstones. Over 90 wells, including exploration and development wells, have been drilled in the area. Seismic amplitude data, including full and partial offset stacked, were analyzed to classify gas, water, and shale zones. The workflow’s performance is demonstrated through the successful identification of the low-amplitude Swan-E Messinian anomaly, characterized as a high-risk gas prospect. Machine learning techniques, specifically neural network models, were trained to differentiate seismic features such as low-amplitude gas sand from background-amplitude water sand and shale. By iterating over multiple attributes and validating the models on blind test sets and on a blind section, which excluded a known shallow gas field, the workflow significantly improved the ability to detect potential hydrocarbon reservoirs characterized by low seismic amplitude. The results show that this integrated approach reduces exploration risk, quantifies the chance of success, and enhances decision-making in well placement and hydrocarbon exploration. This method is particularly useful for identifying low seismic amplitude anomalies, which are often challenging to detect with conventional seismic analysis. (1) This study developed a workflow to detect low seismic amplitude gas fields in near-field exploration. (2) It uses a machine learning algorithm to classify and explore low-seismic-amplitude gas sand reservoirs. (3) This approach helps estimate the likelihood of success and reduces the risk associated with hydrocarbon exploration wells.

## Introduction

The exploration of hydrocarbon reservoirs in deep-water settings presents a range of challenges, particularly in the detection of low seismic amplitude gas fields. These fields, characterized by subtle seismic signatures, are often difficult to derisk using conventional exploration techniques. The success of hydrocarbon exploration in such environments requires innovative approaches to enhance detection accuracy and reduce the risk of drilling unproductive wells. In this context, the integration of advanced seismic analysis methods and machine learning algorithms offers a promising solution.

This study aims to develop a robust workflow to detect non-channelized low seismic amplitude gas fields and define their background amplitude water legs, focusing on the West Delta Deep Marine (WDDM) concession in the northwestern Nile Delta offshore Egypt. Despite the successful exploration of multiple fields within the region, non-channelized low-amplitude gas sands remain challenging to detect due to their subtle seismic expressions. In the Lower Pliocene and upper Messinian, the absence of well data presents an additional issue since all existing well data pertains only to the upper Pliocene.

To address this challenge, the proposed workflow begins by identifying all potential fairways, including low seismic amplitude gas sands and background seismic amplitude water sands. Seismic spectral decomposition is employed to delineate the boundaries of these non-channelized fairways, providing critical insights into their architecture and depositional history. Spectral decomposition has proven effective in revealing intricate geological features such as incised channels, which contribute to a more precise understanding of reservoir distribution.

The workflow incorporates Amplitude Versus Offset (AVO) analysis to validate the gas sand anomalies, focusing on AVO class distinctions between gas sands, water sands, and other facies within the identified fairways by spectral decomposition. These AVO classes are further refined using machine learning algorithms that integrate multiple seismic attributes to classify the entire seismic volume into its most likely facies domains. By leveraging machine learning, the workflow can isolate target features from background seismic, improving the accuracy of anomaly detection.

The overarching goal of this study is to reduce the exploration risk associated with non-channelized low seismic amplitude gas fields and to enhance the ability to predict hydrocarbon prospects with greater confidence. Through the combination of spectral decomposition, AVO analysis, and machine learning techniques, the workflow seeks to quantify the chances of success for exploration prospects with unfamiliar petroleum traps, ensuring more informed decision-making in hydrocarbon exploration projects.

## The study area

**Fig. 1:**
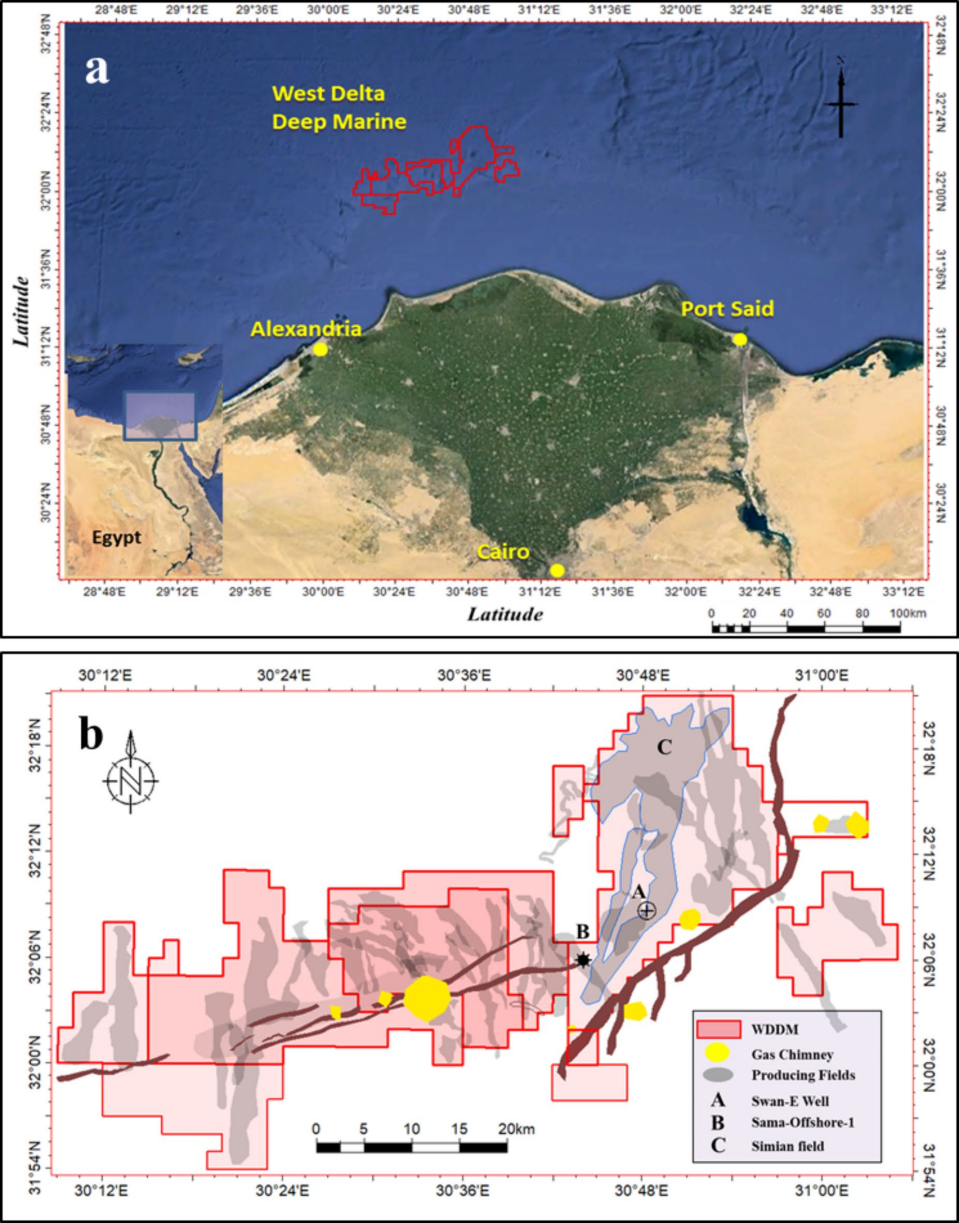
(**a**) A map showing the location of the West Delta Deep Marine concession, modified from Google Earth, and (**b**) The study area location map and the location of Simian field and Sama-Offshore-1 well that were used in the study to de-risk the Swan-E upper Messinian discovery that is also showing in the map. Created in Rashpetco Egypt using Petrel 2023 sofware, www.slb.com.

The West Delta Deep Marine (WDDM) concession lies 50–100 km offshore Alexandria in what is now called the Mediterranean Sea. It covers 1850 km2 of the northwestern margin of the Nile Delta cone, with water depths ranging between 500 and 1500 m, as shown in Fig. 1. The study area covers most of the WDDM’s current important Pliocene fields. In 2010, the US Geological Survey (USGS) estimated 1.8 billion barrels of recoverable oil, 223 trillion cubic feet of recoverable gas, and 6 billion barrels of natural gas still undiscovered across the Nile Delta basin^‎1^.

The reservoirs consist of successions of sandstones and mudstones layered on top of each other in a fining upward composite sequence (they represent a channelized system). Sand bodies occur in laterally amalgamated meandering channels with frontal splays and levees. These channels are interpreted to be the products of deep-water gravity-flow processes. Above a major basal incision surface, the reservoir consists of high net-to-gross sand made up of laterally amalgamated channels. The medial section of the reservoir is more aggradational, with laterally isolated and sinuous channels. Within the upper part of the reservoir, the channels are narrower, straighter, and built of separate channels associated with frontal splay elements, and the less common channels levee. The main channel system is buried by a prograding slope succession that includes sand-sheet lobes^‎2^.

The major structures within the WDDM concession are the northeast-southwest trending Rosetta fault, the east northeast-west northwest trending Nile Delta offshore anticline (NDOA), and the rotated fault blocks towards the northeast of the concession boundary^‎3^. All have been active at various periods during the Pliocene and Pleistocene geologic ages. The depositional geometries of the Upper Pliocene channels are not affected by the major structures, and they can be traced on the map and seismic sections without significant thickening or change in the sedimentation style across these features^4^.

There are more than 20 successful exploration and appraisal wells in the study area, as well as more than 70 development wells. All of them were drilled on strong and clear amplitude supported in Pliocene prospects, which means all fields have soft-kick seismic signatures and are brighter on the far stack, which is a partial stack from an angle of 30° to 40°.

**Fig. 2:**
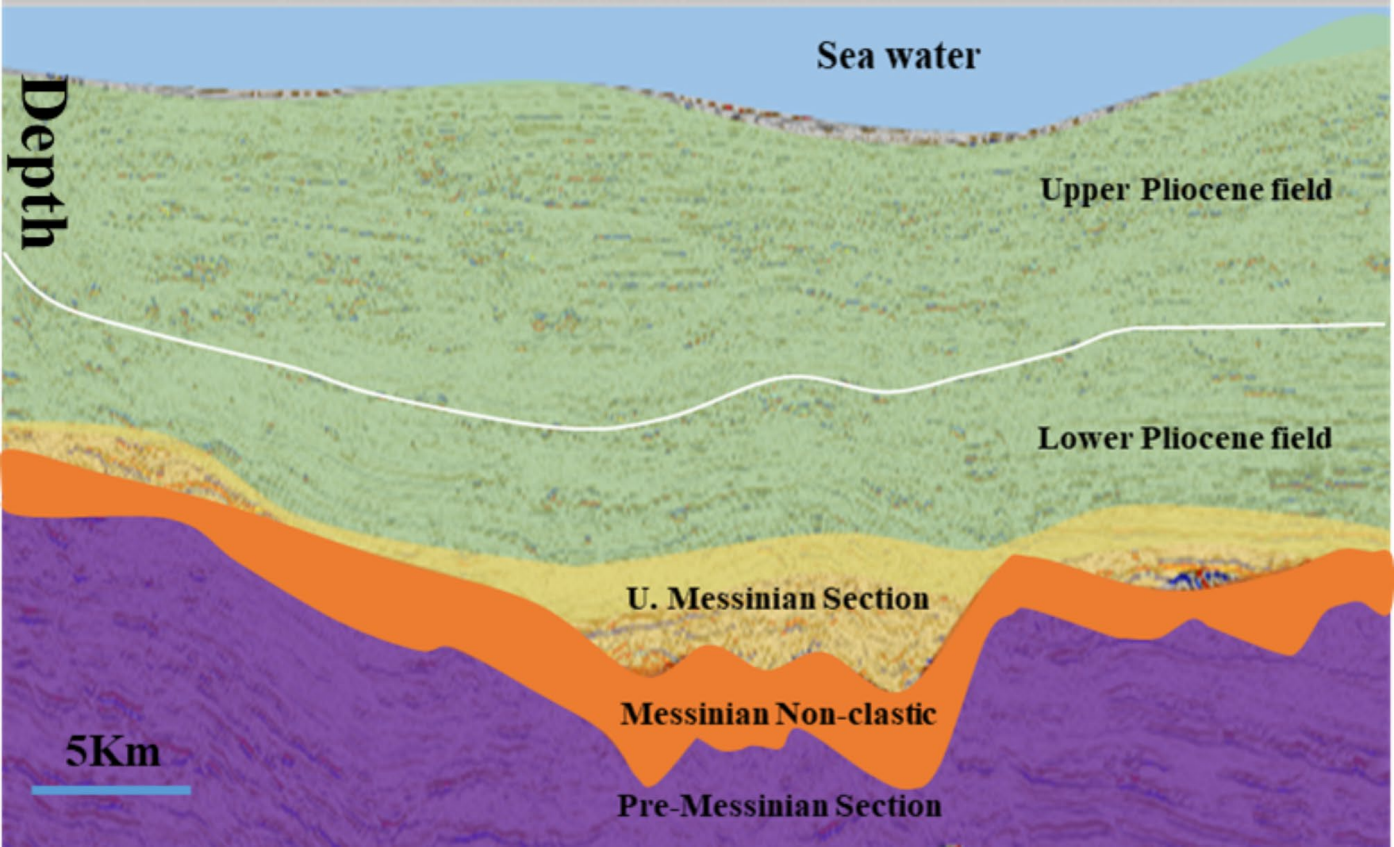
A section showing the stratigraphic sections for the study of the area. The major producing zones are upper and lower Pliocene. There is only one Upper Messinian discovery.

Figure 2 shows the producing zones that range from 1700 to 3000 m TVD BML (below mudline), and all of them are upper Pliocene, lower Pliocene, and **one** upper Messinian discovery (Mina-1) that was discovered in 2006. Mina Field is located on the upthrown side of the Rosetta fault, so it has the same depths as the Pliocene fields that are located in the area. For that reason, the seismic amplitude of Mina field is clearly AVO class-III amplitude supported as Pliocene fields.

In this study, the upper Pliocene Simian field and Sama field were compared to de-risk a low seismic amplitude Swan-E Messinian anomaly (discovery) regarding the amplitude and channelized feature detection, the lithology, and the fluid.

There are also around seven failure cases that were drilled. One of them tested AVO calss-IIp (invisible gas sand) on full stack seismic data in the Pliocene section, and the result was brine sand. Another one was targeting Messinian non-clastic, and the result was no sand. The other **5** wells were targeting the pre-Messinian section and were drilled on geological concepts; only and all of them counted water sand.

This study focuses on low seismic amplitude gas sand in the lower Pliocene and upper Messinian that led to a new upper Messinian discovery, the Swan-E Messinian prospect. The seismic amplitude data used in this study consists of partial and full offset stacked sections with a depth record length of 10,000 m and a sample rate of 5 m. The dominant frequency within the interval of the study is 18 Hz, and the seismic was acquired with the broadband slanted cable technique to benefit from the notch diversity deghosting and to achieve optimal low frequency for deeper targets. The workflow did not require any prior conditioning of seismic data or welllogs. This study used seismic polarity with positive amplitude values for the soft-kick and negative amplitude values for the hard-kick.

### Methodology

### Sand fairway identification and AVO classification

In this study, we used spectral decomposition for sand fairway identification and AVO classification to validate the gas sand part of the fairway. Conventional spectral decomposition is usually performed using the Short Window Discrete Fourier Transform (SWDFT)^5^ proposed the application of the SWDFT to generate common frequency cubes. The concept is to select a data analysis window containing stratigraphic features of interest. The next step is to transform the depth-domain data to the frequency domain using the SWDFT. After spectral balancing, the decomposed common frequency horizon slices were extracted to identify textures and geological patterns. The three spectral components were co-rendered by plotting them against red-green-blue (RGB) color model components^6^.

Spectral decomposition is used to reveal incised channels on the incise slope, basin toe, and large fans in deep water. This may compare favorably against the standard methods of amplitude, phase, frequency extraction, and coherency attributes as it displays the channel image changes over the desired windows of the frequency spectrum. Spectral decomposition can also provide valuable insights into a region’s depositional history. Once the spectral decomposition is performed, unsuspected features are revealed that may contribute further insight and understanding with regards to reservoir mapping and the delineation of stratigraphic and structural features such as channel sands and complex fault systems^7^.

**Fig. 3:**
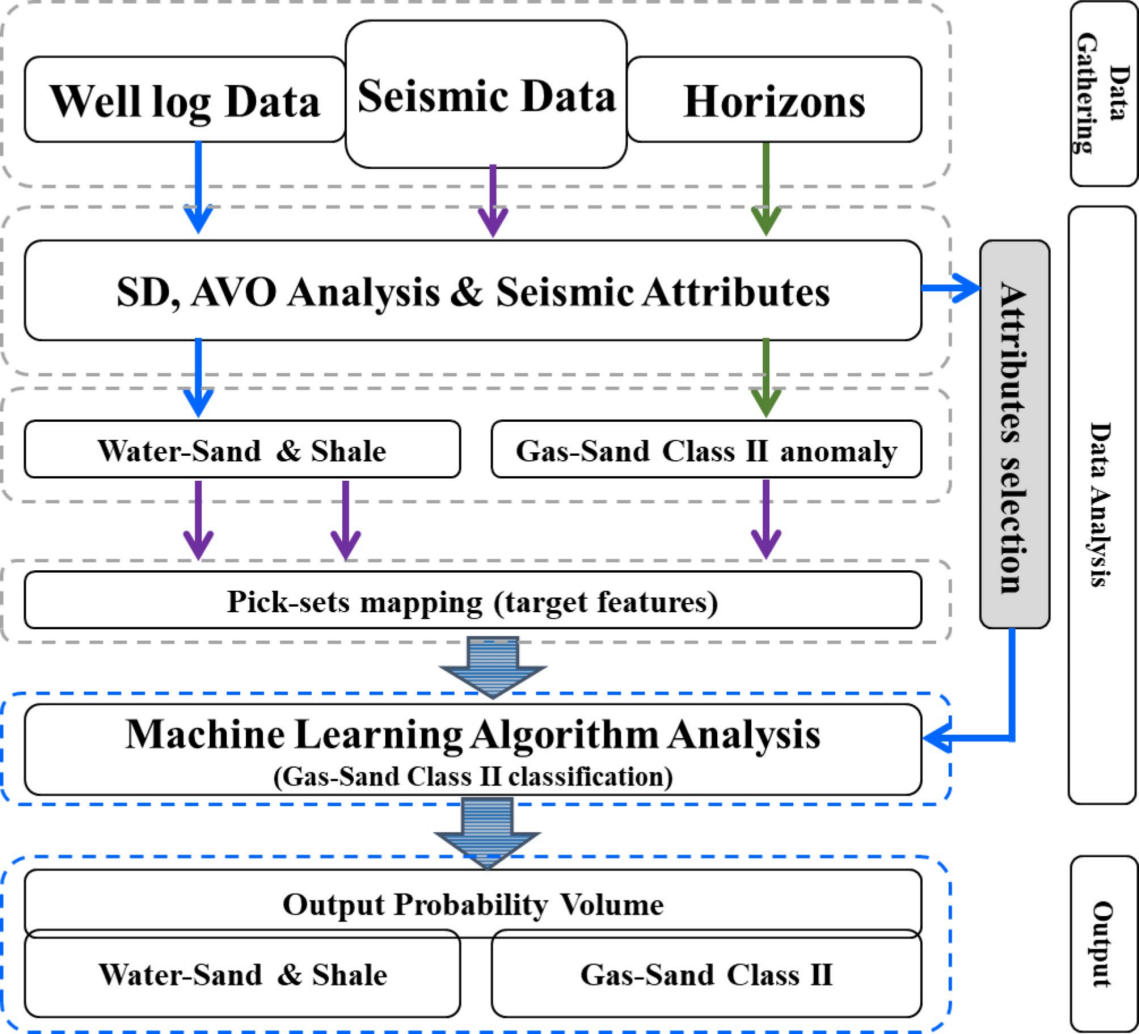
A workflow diagram showing the detailed workflow steps for the gas sand low seismic amplitude AVO class-II Cube approach. The seismic data is used as input after deriving a suite of attributes and then validating one anomaly as gas sand low seismic amplitude using AVO analysis. After that, the machine learning algorithms used to convert the input seismic to represent the gas sand AVO class-II, water sand, and shale zones and ultimately produce a gas sand AVO class-II cube as output.

**Fig. 4:**
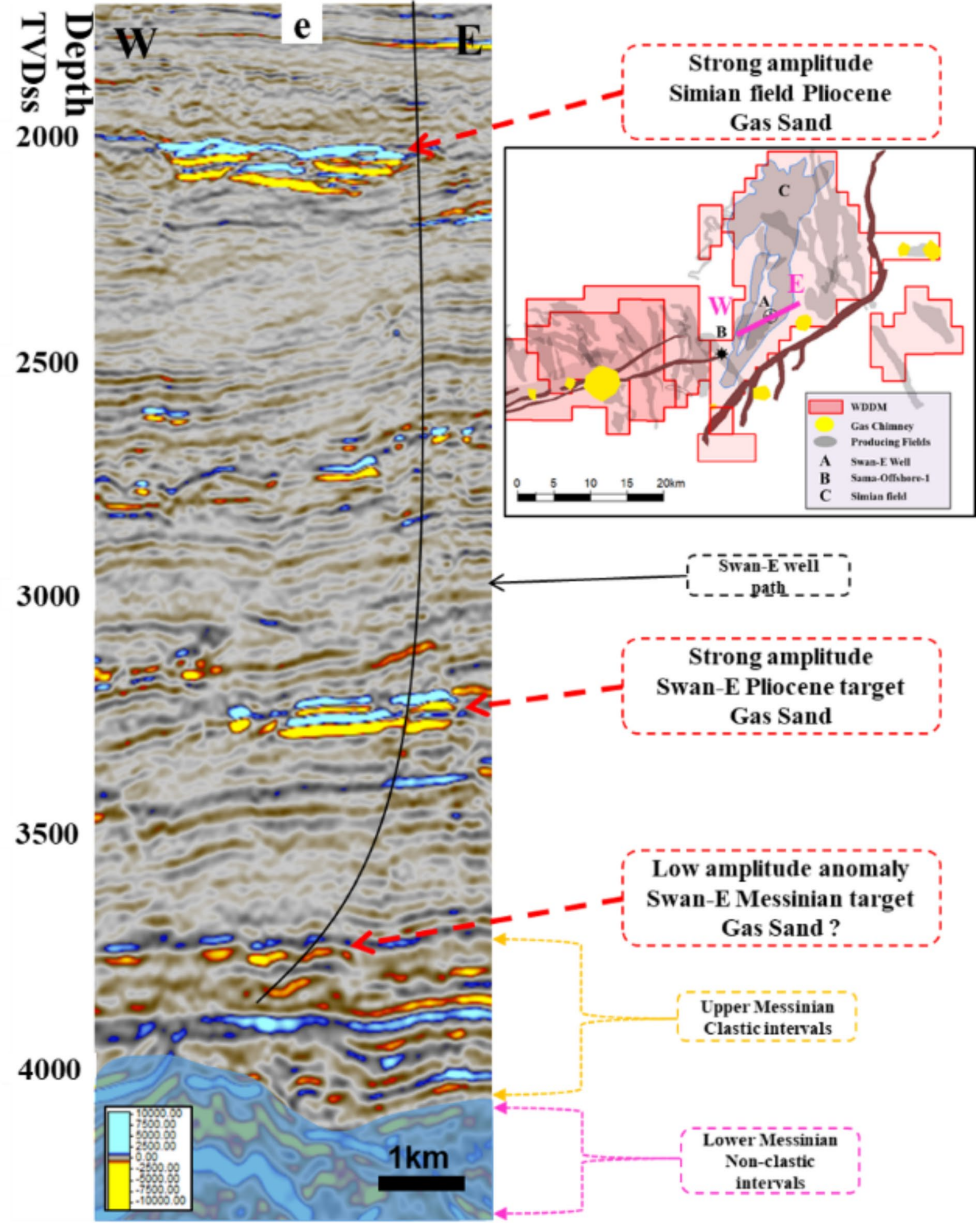
A randon cross section shows a Swan-E well path that penetrates a strong amplitude soft-kick Swan-E Pliocene target and penetrates a very low seismic amplitude soft-kick Swan-E Mesinain discovery. Created in Rashpetco Egypt using Petrel 2023 sofware, www.slb.com.

**Fig. 5:**
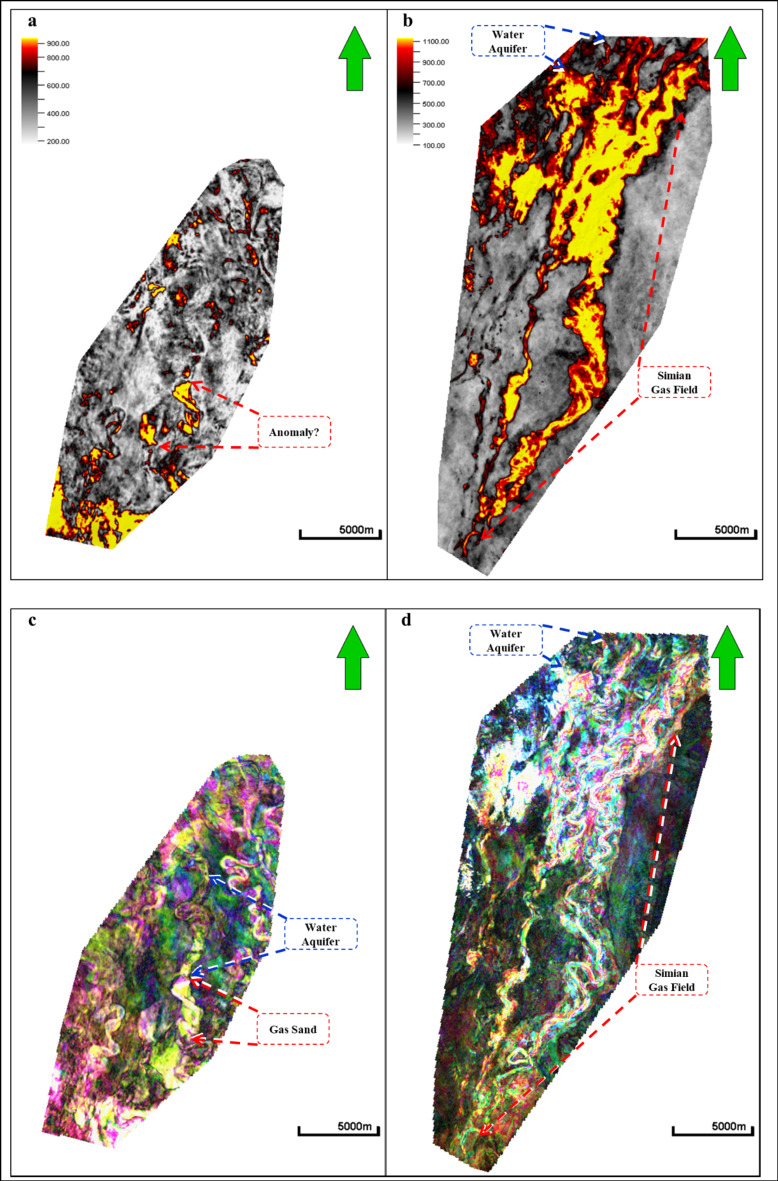
(**a**) A 2D average amplitude attribute map for low seismic amplitude Swan-E discovery and it is not clear channalized feature; oval shape; and (**b**) for upper pliocene Simian produced field channelized features. (c) 2D horizon-based spectral decomposition RGB extraction on low seismic amplitude Swan-E Messinian with clear north fairway; north aquifer; and (d) for upper pliocene Simian-produced field. Created in Rashpetco Egypt using Petrel 2023 sofware, www.slb.com.

**Fig. 6:**
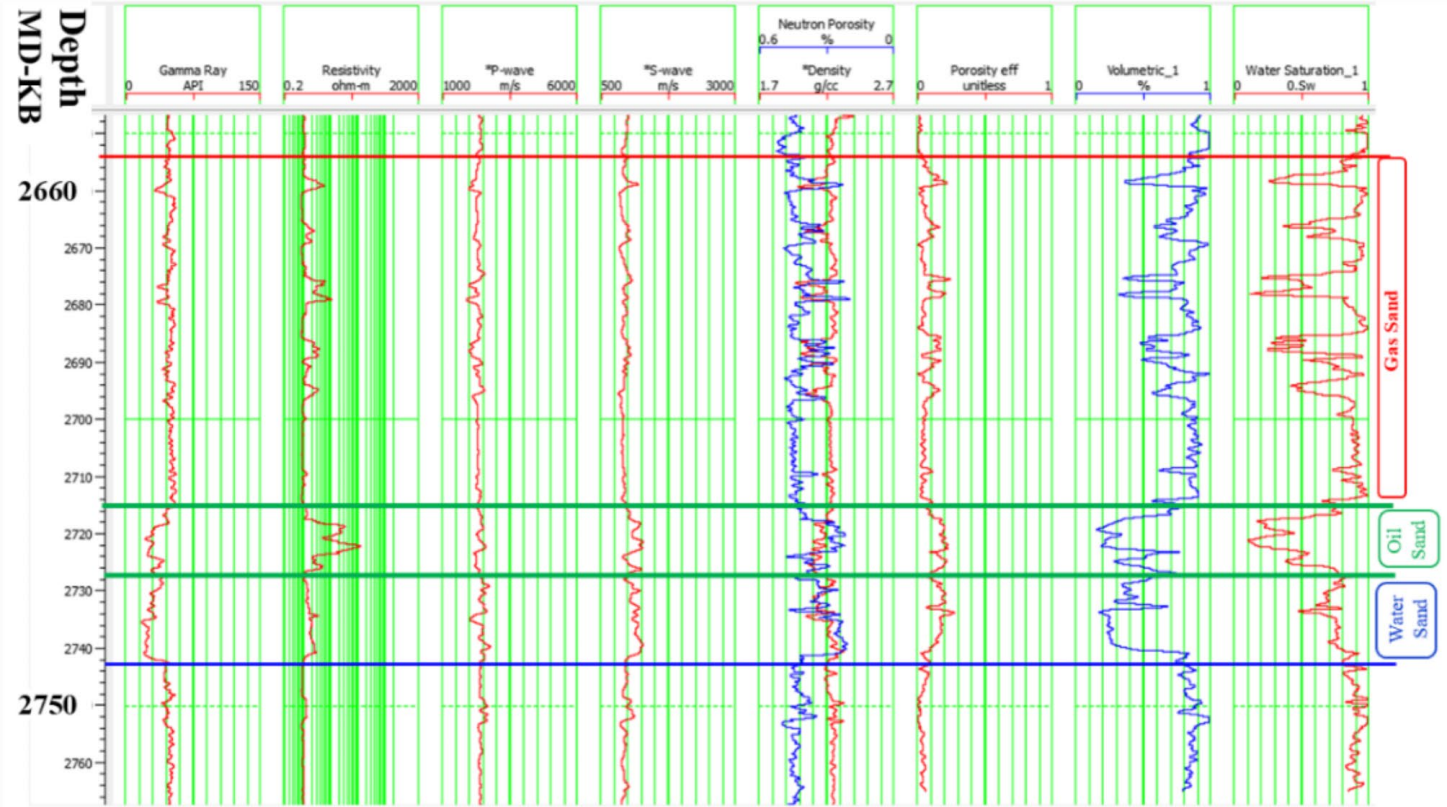
Well logs of Elwastani Formation at the Sama well location and the sand reservoir section.

**Fig. 7:**
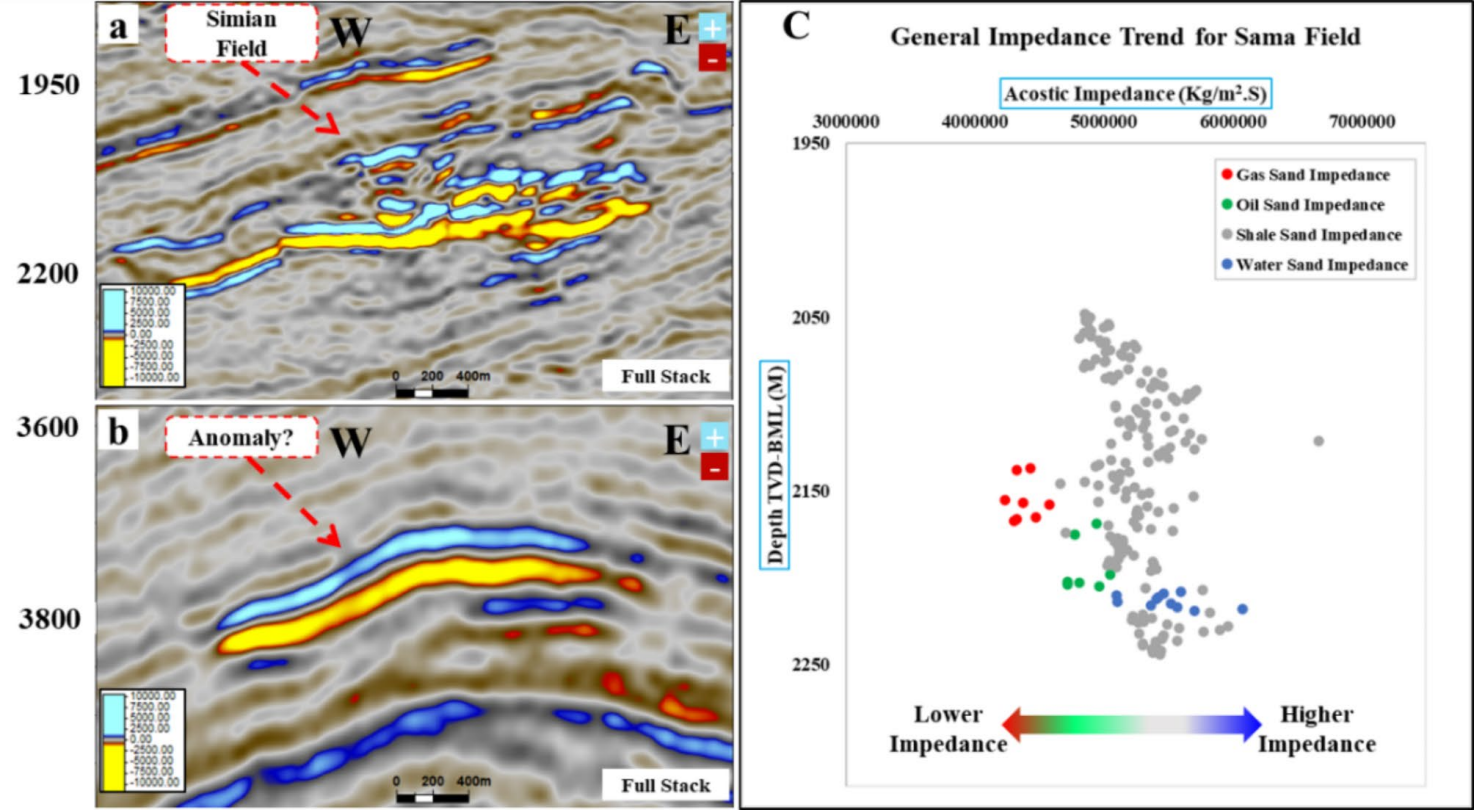
(**a**) A random seismic cross section full stack seismic signature shows for Upper Pliocene Simian produced field seismically multi loops, and (**b**) A random seismic cross section full stack seismic signature shows the soft-kick of the low seismic amplitude Swan-E Messinian discovery seismically single loop. (**c**) A general impedance-depth TVD BML crossplot for Sama Field shows the general impedance trend in the area of the gas sand that is softer than the shale background and the water sand trend that is harder than the shale background. Created in Rashpetco Egypt using Petrel 2023 sofware, www.slb.com.

The procedure involves two main steps, as shown in Fig. 3:

1. Process a spectral decomposition on full-stack seismic. A Fourier transform was used with a 28 m window length and processed three spectral components at 9, 12, and 27 Hz. Next, a screening of the horizon using RGB color model components was performed. Then, a 2D horizon-based extraction was performed over these spectral components on the horizon (surface) of the anomaly and the Simian Pliocene field, and they were compared with the extraction of the average amplitude surface-based. Figure 4 shows a random cross section on a strong amplitude soft-kick simian field at a depth of 2000 m, a strong amplitude soft-kick Swan-E Pliocene 1st target, and a very low seismic amplitude soft-kick Swan-E Messinian 2nd target of the well Swan-E. Detecting anomalies on a random line through the low seismic amplitude gas sand is difficult due to the low seismic amplitude in comparison to the strong shallow gas sand amplitude. Figure 5 shows the average amplitude attribute maps for the low seismic amplitude “Swan-E Messinian” discovery, and it’s appeared as an oval shape trending north east to south west, **not** a clear channelized feature and interpreted before as a high-risk local gas hazard anomaly. The upper Pliocene Simian-produced field and its clear channelized system have many meander channels like the other produced fields in the area. Figure 5 also shows the 2D horizon-based spectral decomposition RGB extraction on the low seismic amplitude Swan-E discovery, and the north fairway is now clear and appears as a channelized feature running from the south to the north, as that north fairway completes the picture of the channelized feature. On the right-hand side is the 2D horizon-based spectral decomposition RGB extraction on the upper Pliocene Simian-produced field.

2. AVO classification for the Swan-E Messinian prospect. Following successful fairway detection, an AVO investigation was required to understand the anomaly’s lithology and fluid. The Sama-Offshore-1 well was used for AVO classification in addition to the Simian Field. The Sama field is the nearest one to the anomaly and has gas sand, oil sand, and water sand (Fig. 6). There are three AVO investigation steps^8‎,9^ required to de-risk a low seismic amplitude anomaly. The first is the soft-kick seismic response of the anomaly on the near stack or zero angle (intercept) and on the full stack or far stack Fig. 7. The soft-kick investigation has a reason that comes from the general impedance trend in the area of investigation that shows the gas sand is softer than the shale background and the water sand is harder or equal to the background Fig. 7. The seismically single loop is increasing the risk of the anomaly analysis, which is also interpreted as low gas sand facies, low porosities, low net sand, or low gas saturation. For that, the second step is to check the increasing amplitude with increasing offset as an indicator for hydrocarbon due to the effect of lower shear velocity of hydrocarbon sand at non-zero offset of the seismic^10–13^.

**Fig. 8:**
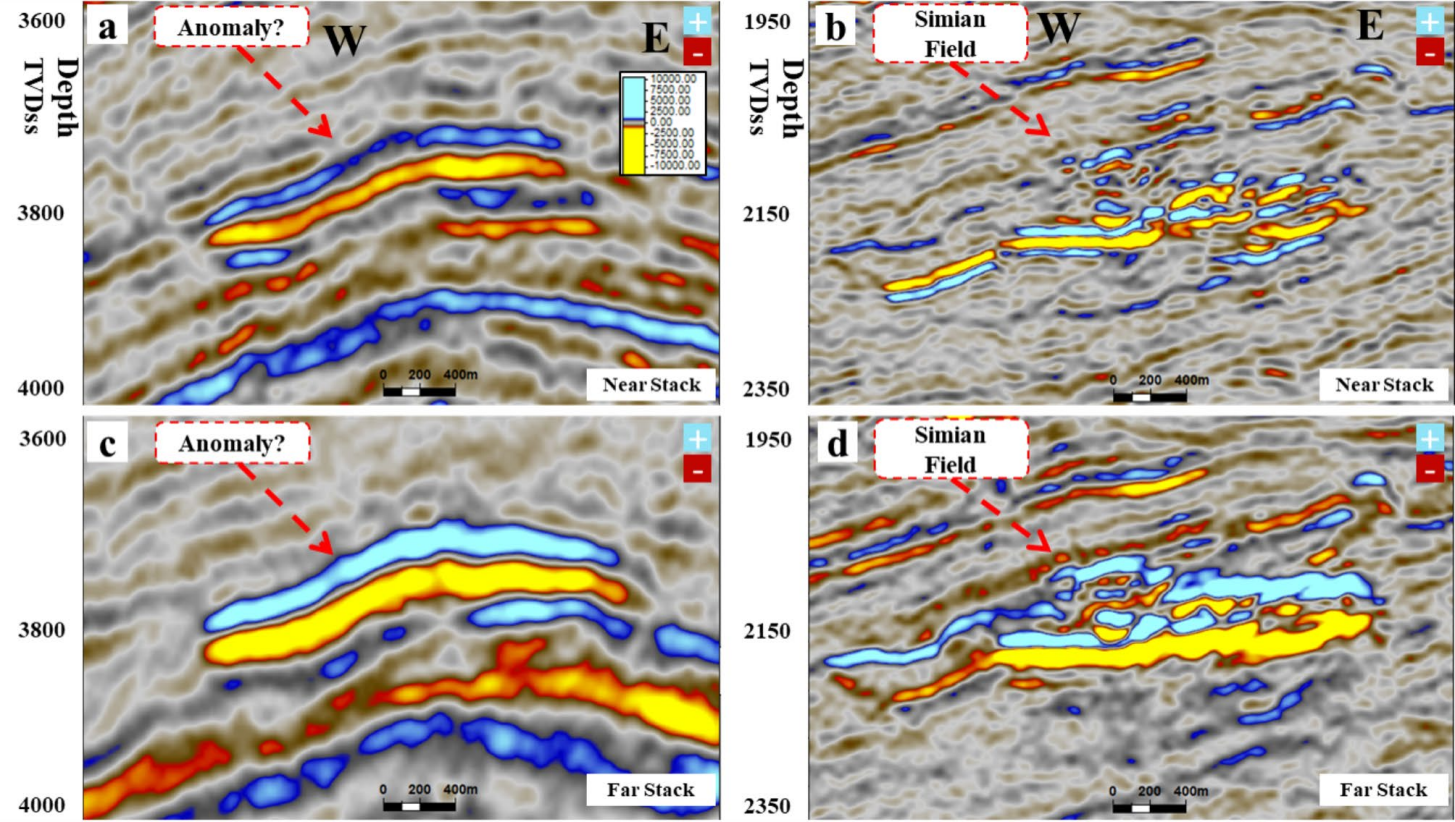
(**a**) A random seismic cross section near angle stack from 10°-20° shows the low seismic amplitude Swan-E Messinian discovery, and (**b**) for the Upper Pliocene Simian-produced field. (**c**) Far angle stack from 30° to 40° for low seismic amplitude Swan-E Messinian discovery, and (**d**) for Upper Pliocene Simian produced field seismically multi loop.

**Fig. 9:**
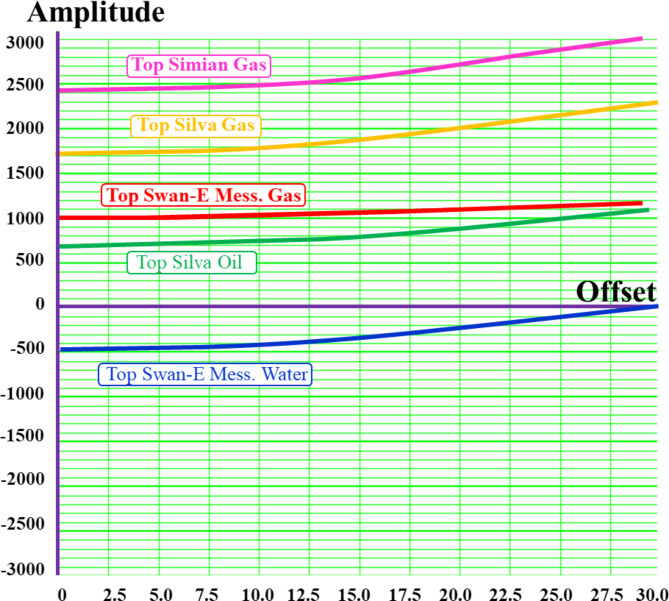
(**a**) AVO analysis shows the amplitude (y-axis) variation with angles (x-axis) and indicates the increase of the amplitude with the angles, and the intercept of the Swan-E Messinian is lower than the gas sand of the Simian and Sama Field and higher than the intercept of the oil sand of the Silva Field, while the Swan-E Messinian is deeper than the Simian field by 2 km and the Sama field by 1 km.

Figure 8 shows the random cross section of the near-angle stack for the SwanE Messinian and for the Simian field and their far-angle stack, which is brighter than the near-angle stack. This amplitude brightening of the far-angle stack is a hydrocarbon indicator. The intercept of the anomaly was compared to the Sama Field, which has gas sand and oil sand. Figure 9 shows that the analysis of the intercept of the Swan-E Messinian discovery is higher than the intercept of the oil sand of Sama-Offshore-1, and it is an indicator that the Swan-E Messinian is most probably gas as it’s deeper than Sama-Offshore-1. If the intercept of the Swan-E Messinian discovery is lower than the intercept of the oil sand of Sama-Offshore-1, it could be oil sand or gas sand. Regarding the water sand, is a faint hard-kick with decreasing the amplitude with the offset to zero or a very faint soft-kick at high offset, as shown in Fig. 9. The water sand at the lower Pliocene level and upper Messinian level cannot easily be detected on full stack seismic or its average amplitude.

**Fig. 10:**
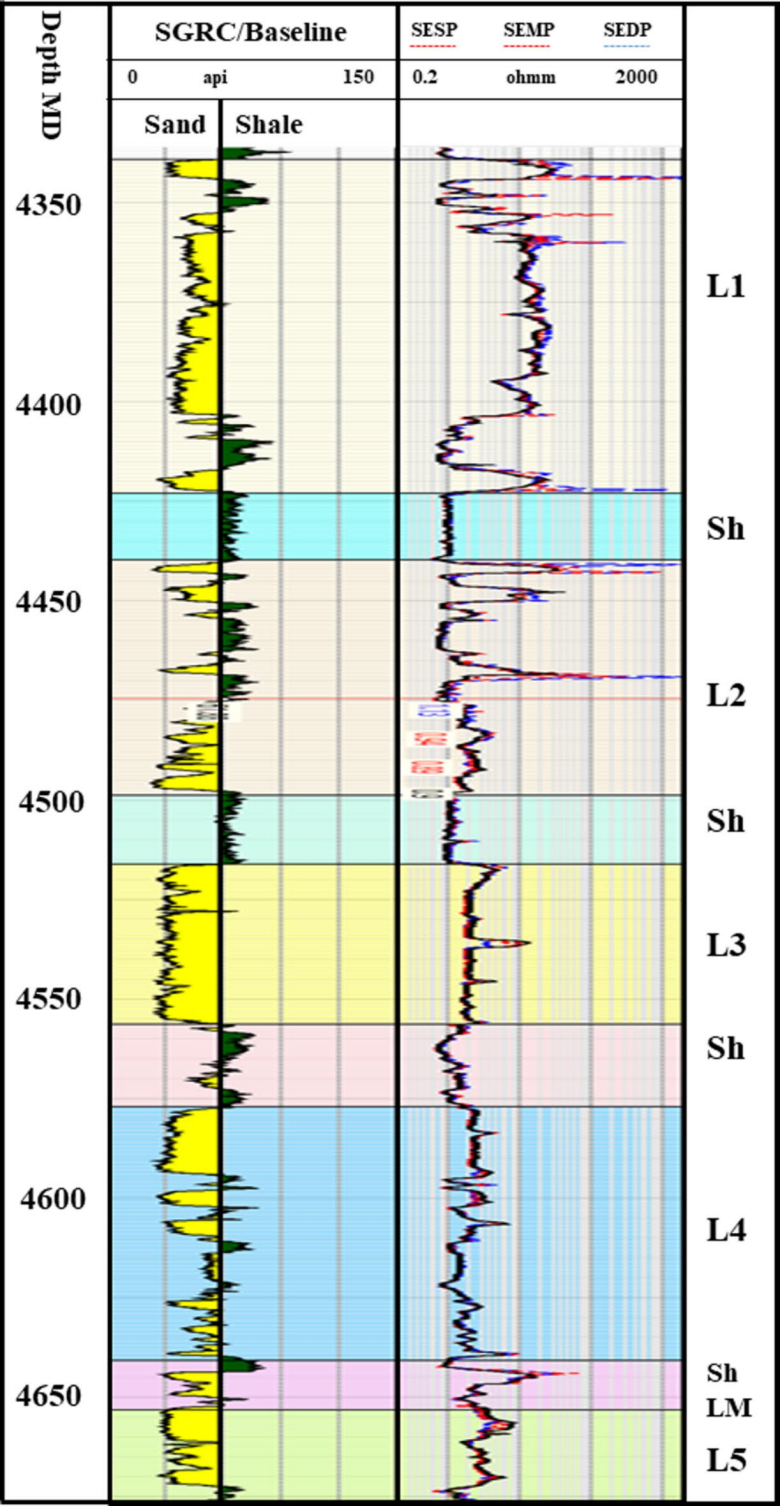
Show a LWD for Gamma Ray and Resistivity for SwanE Messinian Discovery with 124 m MD and 74 m TVD gross pay from 247 m MD and 330 m TVD gross thickness.

The third step is to extend the investigation of the second step for all the anomalies, not just one point. For that, 3D seismic data was used to create spectral decomposition volume attributes (Fig. 5). This spectral decomposition makes it clearer that a bright anomaly is related to hydrocarbons. It is a better way for hydrocarbon prospects screening in the whole area, while spectral decomposition on the seismic full stack volume is necessary for the fairway detection process. For all previous investigations, the SwanE Messinian well was drilled and resulted in gas sand within the first two levels, L1 and L2. Figure 10 shows five sand intervals separated by four shale intervals and the gas sand filled with L1 and L2. The gross pay is 124 m MD and 74 m TVD from a gross thickness of 247 m MD and 330 m TVD, with porosity up to 27% and gas saturation around 71%.

### Identification of AVO Class II using machine learning

A machine learning technique is a mathematical algorithm that can be trained to solve a problem that would normally require human guidance^14^. The main advantage of machine learning over most traditional estimation methods is their ability to determine a nonlinear relationship between seismic properties and well log properties or any features that can be detected on the seismic data, such as gas chimneys, salt domes, faults, ridges, slumps, etc. By generating seismic attributes physically related to the reservoir properties and combining them, we can predict the petrophysical properties of the reservoir^15–17^. Either multi-linear regression or machine learning analysis can achieve the combination of the attributes. An extrapolation for the identified seismic facies classes (e.g., gas sand, water sand, shale) throughout the seismic volume was achievable once a relationship was established between the seismic attributes and the target classes.

Once a single instance of low seismic amplitude gas sand in the AVO class-II prospect is verified, machine learning techniques were applied to detect and differentiate similar seismic characteristics associated with low seismic amplitude gas sand from water sand and other backgrounds such as shale^18–22^.

**Fig. 11:**
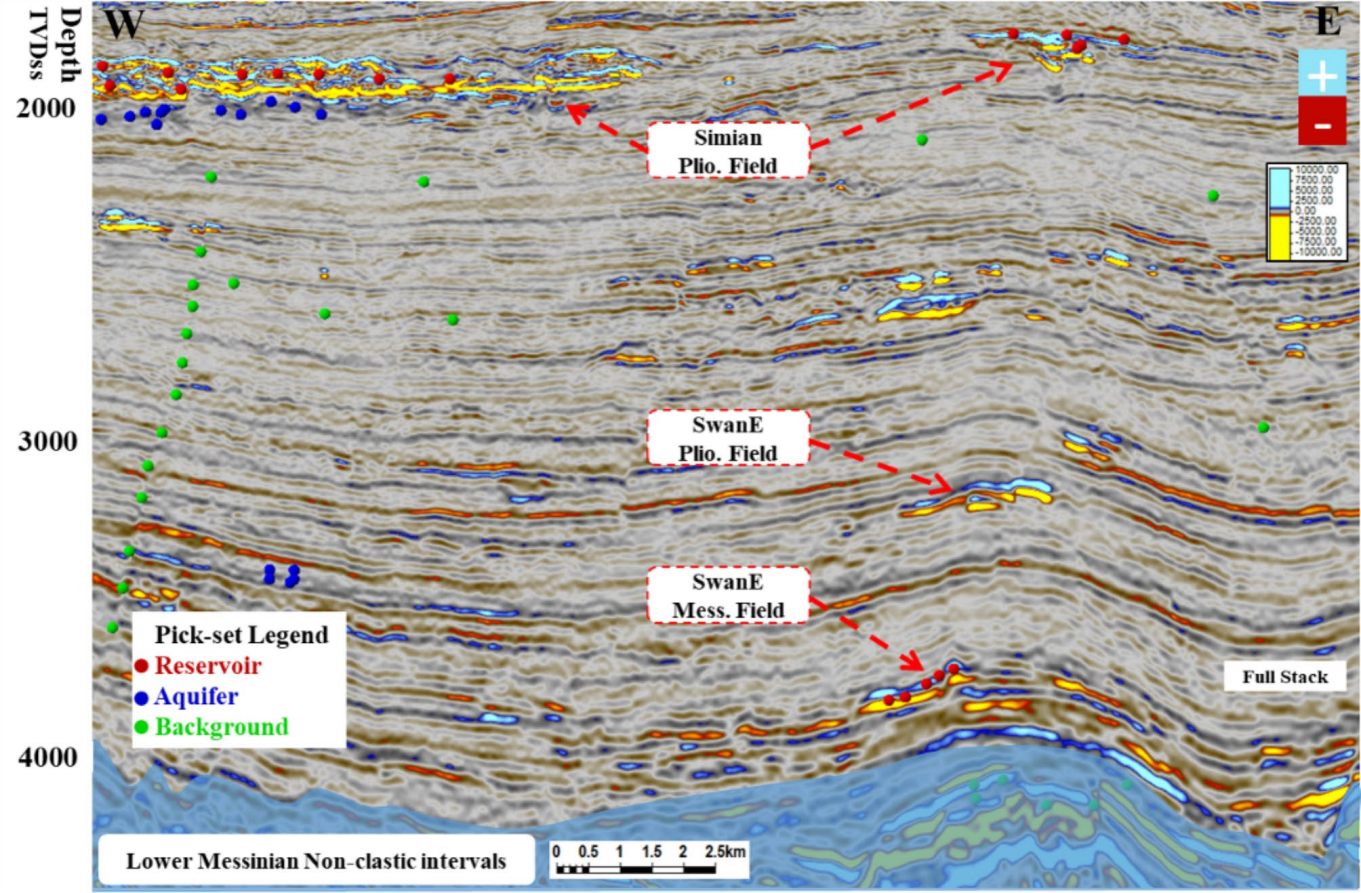
Random cross section shows examples of the gas sand low seismic amplitude AVO class-II (red) picks, water sand (blue) picks, and shale background (green). The green picks cover most of the shale background locations (high and low frequency, faults, anomalies, etc.).

The procedure involves four main steps^23^, as shown in Fig. 3, with OpendTect software:

1. Mapping of the target features (gas sand, including low seismic amplitude confirmed examples, water sand, and shale) as seed points to use them with input seismic data far-angle stack. These seeds have x- and y-coordinates and depth values, and they are termed the ‘pick-set’; the gas sand pick-set is a number of points representing gas sand in the seismic data far-angle stack. The pick-sets for all known water sand and shale are also required. Figure 11 shows an example of the gas sand low seismic amplitude AVO class-II and high amplitude AVO class-III (red), water sand picks (blue), and shale picks (green). The pick-sets serve to indicate how the neural network should discriminate between the characters found in the input attributes. The gas sand cube is crucial for identifying subtle gas sand abnormalities that may not be easily discernible on seismic data or its properties. The presence of low seismic amplitude gas sand anomalies in conjunction with a non-clear water aquifer can provide significant potential for unexplored opportunities.

Identifying or reducing the risk associated with these objectives is often challenging using the standard full-stack seismic reflection dataset. This is because they have either a similar amplitude to the background or an amplitude resembling unfavorable characteristics such as poor rock quality, low porosity, low hydrocarbon saturation, or a local gas hazard anomaly. Consequently, mapping and mitigating the risk associated with these objectives is difficult.

2. Selecting the input seismic attributes and extracting them from the input seismic cube far-angle stack. The input attributes should be physically related to the target of the picks. During the training stage of the neural network, the weights of the attributes were recorded, and these weights indicate the relative importance of each attribute for the classification of the targets. A lot of iterations were run to achieve the best attribute list to drive the reservoir, aquifer, and background cubes. The final attribute list was successfully created after many iterations to select the highest weights and tabulated in Table 1. Thirteen attributes were utilized and expanded to twenty-nine attributes, with various parameters meticulously selected after iterations to achieve the highest weights.

**Table 1:** The attributes list for gas sand low seismic amplitude workflows and the role of each one to distinguish between the low seismic amplitude gas sand zones from waster and shale zones.

Attribute List
Energy
Event
Average Frequency
Frequency Slop Fall
Instantaneous Amplitude
Instantaneous bandwidth
Perpendicular Dip Extractor
Semblance
Texture
Frequency Beyond
Instantaneous Frequency
Sweetness
Similarity

**Fig. 12:**
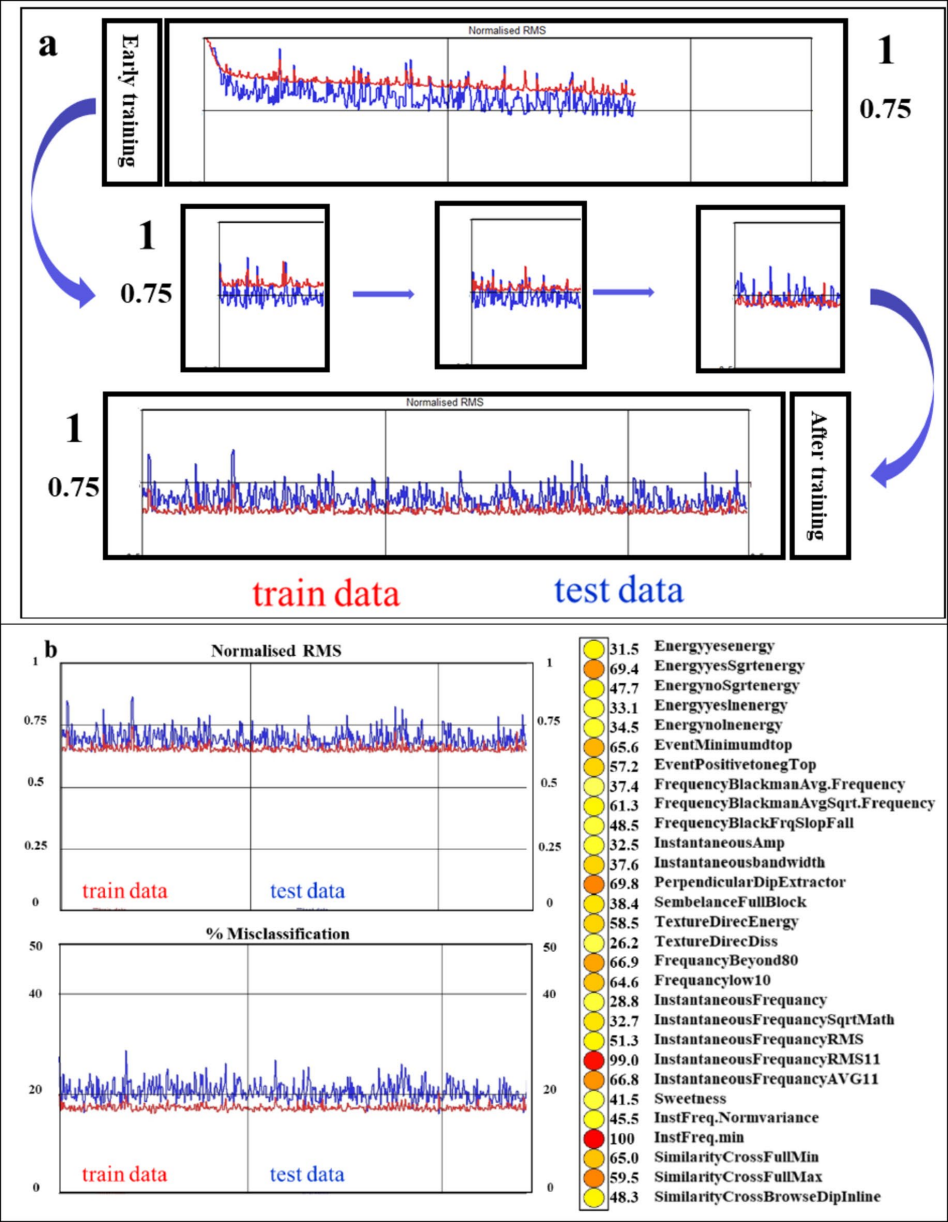
The training of a low seismic amplitude gas sand AVO class-II machine learning neural network. (**a**) The behavior of the data and test curves during model training. (**b**) The left hand side displays the normalized RMS and misclassification results. The right-hand side shows the relative importance of nodes (all input attributes that are used in the workflow); the dark red nodes (higher values) are the most important ones.

**Fig. 13:**
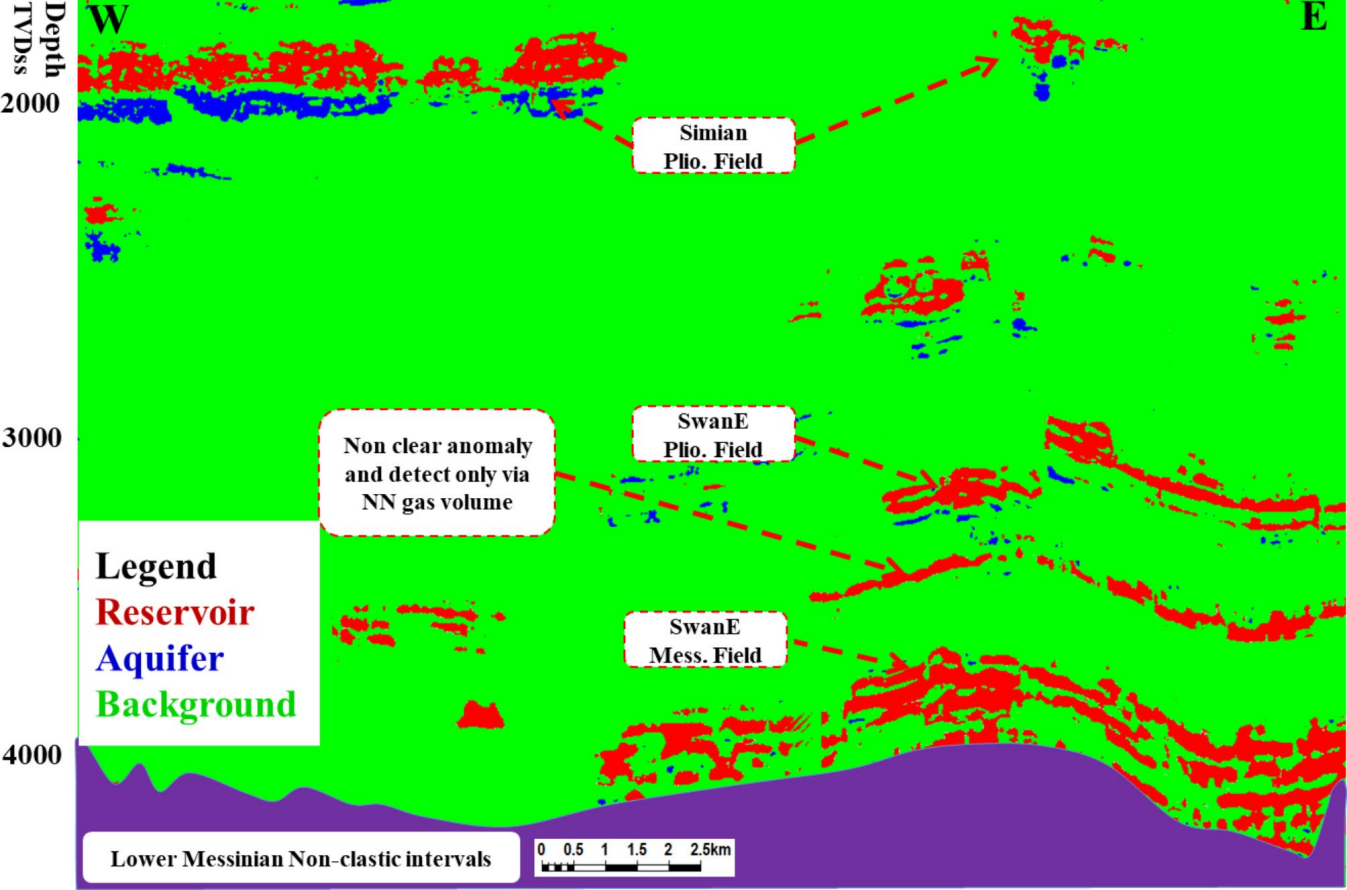
Random cross section shows the result of gas sand cube (red), water sand cube (blue), and shale background (green). This cross section through the Simian, Swan-E Pliocene, and Messinian Fields.

3. The training of the designed neural network was started. This methodology employs a supervised neural network featuring 14 nodes in the hidden layers. During the machine learning training, the previously made picks are fed to the machine learning network as an input vector of attributes to classify (gas sand, water sand, and shale). The machine learning is then optimized by back-propagation of the error. The backpropagation algorithm for multilayer networks is a generalization of the LMS algorithm, and both algorithms use the same performance index mean square error. During the training phase, it is very important to find the proper stopping point, as overtraining may otherwise occur. Overtraining occurs when the neural networks find relations in the training examples that are not universal. One way of preventing this from happening is to randomly extract a number of examples from the interpreter-provided training picks and exclude these from the neural network while still evaluating the prediction error for these points, which were referred to this as the “test set error.” The quantity of utilized training input vectors is 3,075,000, while the quantity of employed test data vectors is 879,971. The selected technique, which was used to obtain the gas sand, including low seismic amplitude AVO class-II probability volume, is the supervised classification multi-layer perceptron machine learning neural network. In the supervised mode, network performance is tracked during training in two ways: normalized RMS and percent misclassification. The normalized RMS error curves indicate the overall error on the trained and test sets, in red and blue, respectively, on a scale from 0 (no error) to 1 (maximum error). During training, both curves should exhibit a decline in value. When the test curve ascends and intersects with the data set, subsequently exceeding it, the training should be stopped. Typically, an RMS value in the 0.8 range is considered reasonable; between 0.8 and 0.6 is good; between 0.6 and 0.4 is excellent; and below 0.4 is near perfect. The previous ranking was quoted from the OpendTect software manual. Figure 12 shows the training of the gas sand low seismic amplitude neural network. The left side is the normalized RMS and misclassification, and the right side is the relative importance of nodes (all input attributes that were used in the workflow); the dark red is the highest importance, and the total misclassified for training status is 17.8% and for test status is 19.6%.

4. Apply the trained machine learning network to the seismic data far-angle stack volume to obtain the resulting low seismic amplitude gas sand AVO class-II probability cube based on each sample similarity to the input picks. Figure 11 represents an example of low seismic amplitude gas sand AVO class-II in the seismic section, water sand, and shale background, and Fig. 13 represents the result of identifying low seismic amplitude gas sand, water sand, and shale background at the same section.

## Results and discussion

The Swan-E Messinian anomaly, characterized by a relatively tiny localized oval shape, exhibits a low seismic amplitude of soft-kick compared to a vast channelized incised system. However, it demonstrates a strong soft kick relative to the seismic background. As a result, this anomaly has been avoided for drilling purposes for over 20 years due to its high-risk ranking. To demonstrate the fairway of the prospect Fig. 5, the spectral decomposition was performed on the Swan-E Messinian anomaly. This fairway was not visible on the seismic or surface features. The AVO study was used to mitigate risk and determine the lithology and fluid properties of the anomaly. As a result, the amplitude of the anomaly increased with the offset, and the intercept of the anomaly was higher than the oil found in the Sama field. The preceding investigation has determined that the anomaly has transformed into a channeled feature containing gas sand and the northern water aquifer.

The machine learning neural network back-propagation algorithm was used to identify the same anomalies: low seismic amplitude gas sand AVO class II. The output cube is a 3D cube that has two values: 1.0 at the gas sand, including low seismic amplitude gas sand AVO class-II locations, and 0.0 at the non-gas sand locations. The water sand cube is a 3D cube that has two values: 1.0 at the water sand and 0.0 at non-water sand. The last cube is a 3D shale cube that has two values: 1.0 at the shale background and 0.0 at the non-shale background.

**Fig. 14:**
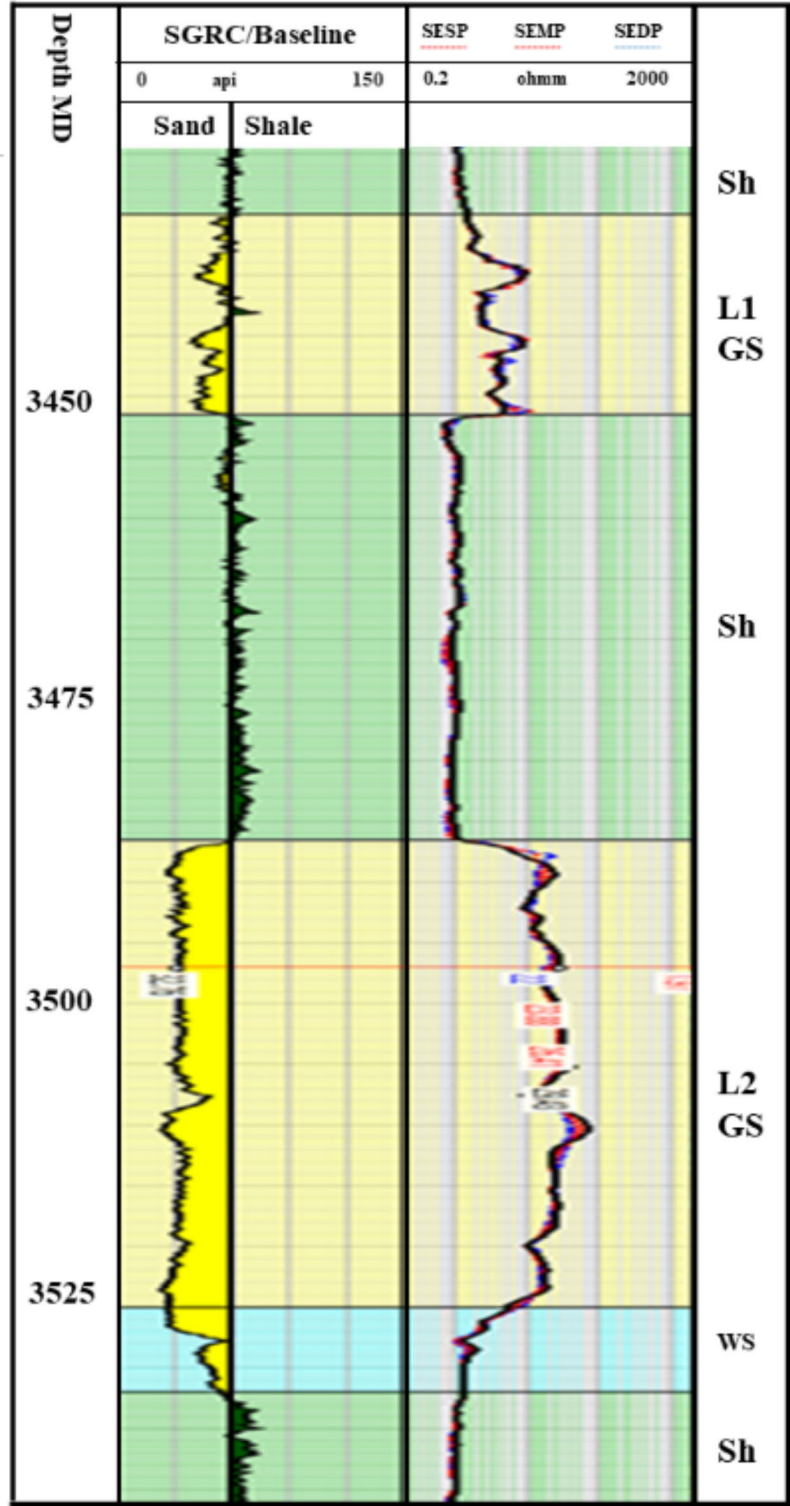
The shallow Swan Pliocene field gas sand markers on Swan-E from 3435 to 3525 m MD and this shallow interval were not included in the input data and were used as ‘blind’ data for validation and QC.

**Fig. 15:**
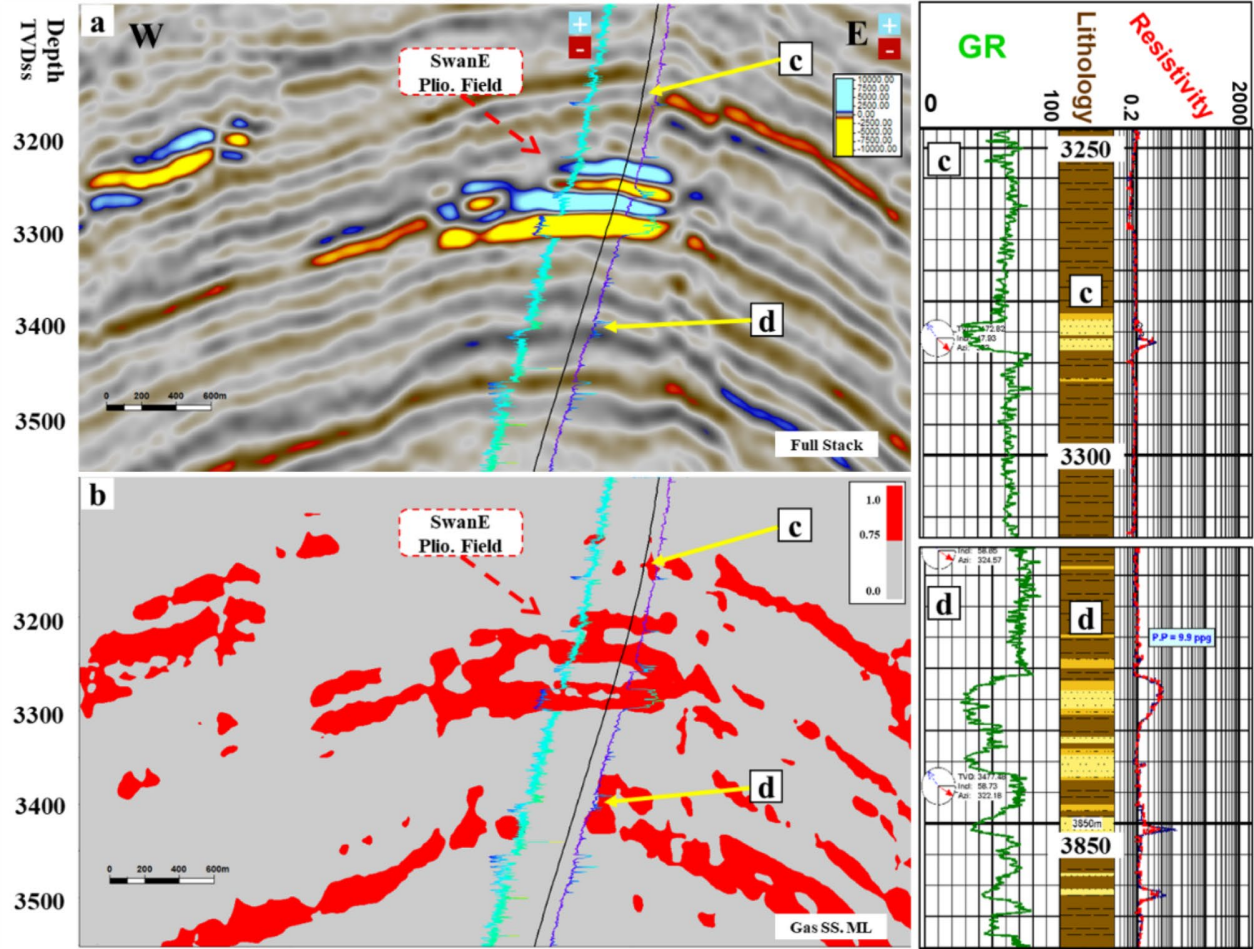
(**a**) The seismic cross section on SwanE Pliocene field; (**b**) ‘Gas sand cube’ and reveals the shallow Swan pliocene field, although its pickset is not included in the input (gas sand in red, water sand, and shale in gray); (c) and (d) are two composite logs for two low seismic amplitude gas sands not clear on the seismic cross section and detectable on the gas sand cube.

The best case for validation is testing the results using a ‘blind’ section. In our scenario, we excluded the shallow Swan Pliocene field from the study. Swan-E Pliocene field also has a shallow gas sand AVO class-III, which was drilled before by two wells. Swan-E well penetrated the shallow Pliocene gas field at depth range 3435 to 3525 m MD (equivalent to 3264.06 to 3311.18 m TVD), as shown in Fig. 14. Note the high values in the resistivity log, which indicate gas sand. Figure 15 shows the seismic full stack cross section and the gas sand cube, revealing potential gas sand in the Swan-E Pliocene field. However, this information was not included in the machine learning procedure. Figure 15 shows two composite logs representing two gas sands and corresponding seismic section and gas sand cube response. The gas sand (d) has low seismic amplitude and has indistinct amplitudes on the seismic cross section but can be detected on the gas sand cube. This serves as strong evidence for the sensitivity of amplitude detection in low seismic amplitude gas sands.

**Fig. 16:**
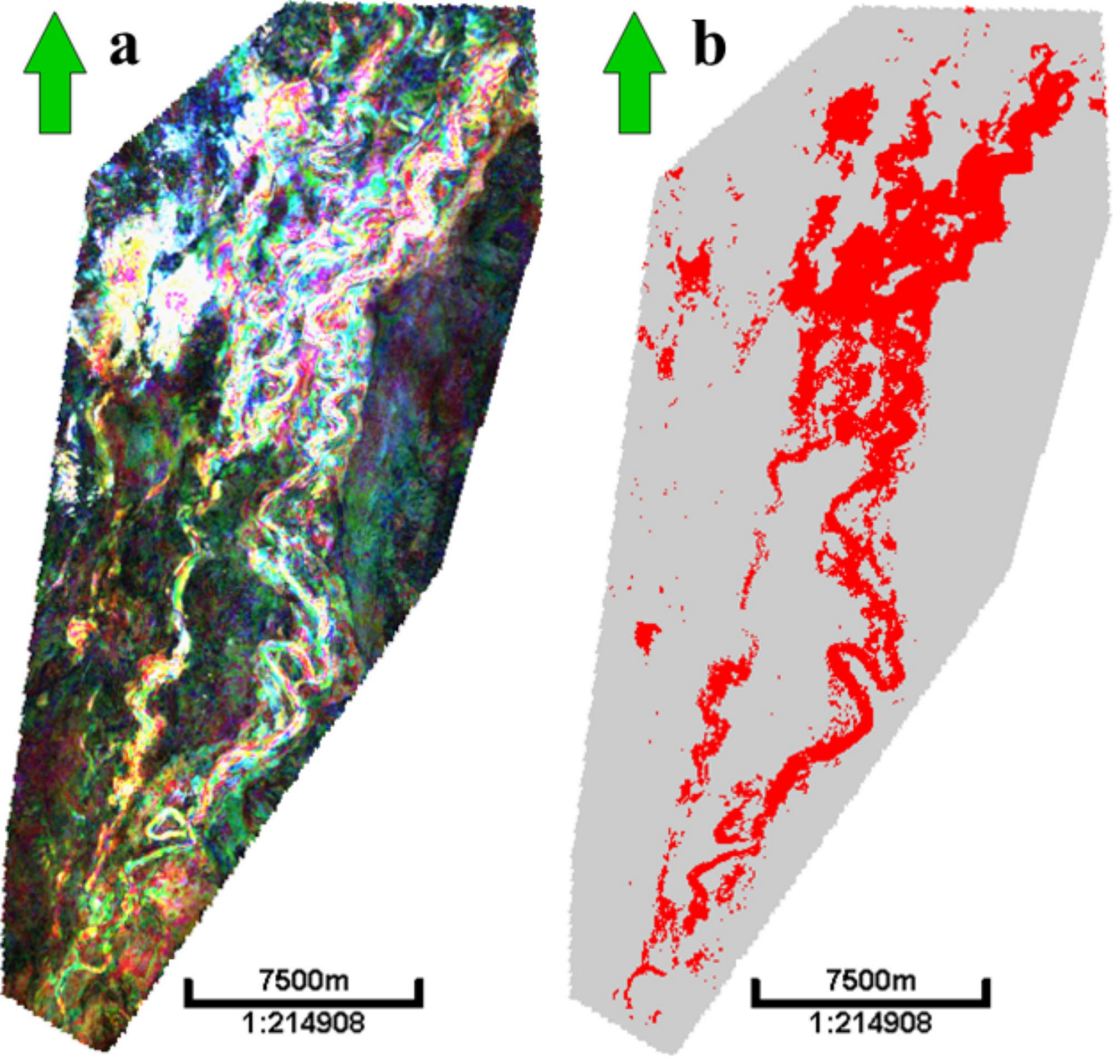
(**a**) 2D slice of spectral decomposition for Simian field, and (**b**) shows a 2D slice of a gas sand cube for Simian field. Created in Rashpetco Egypt using Petrel 2023 sofware, www.slb.com.

**Fig. 17:**
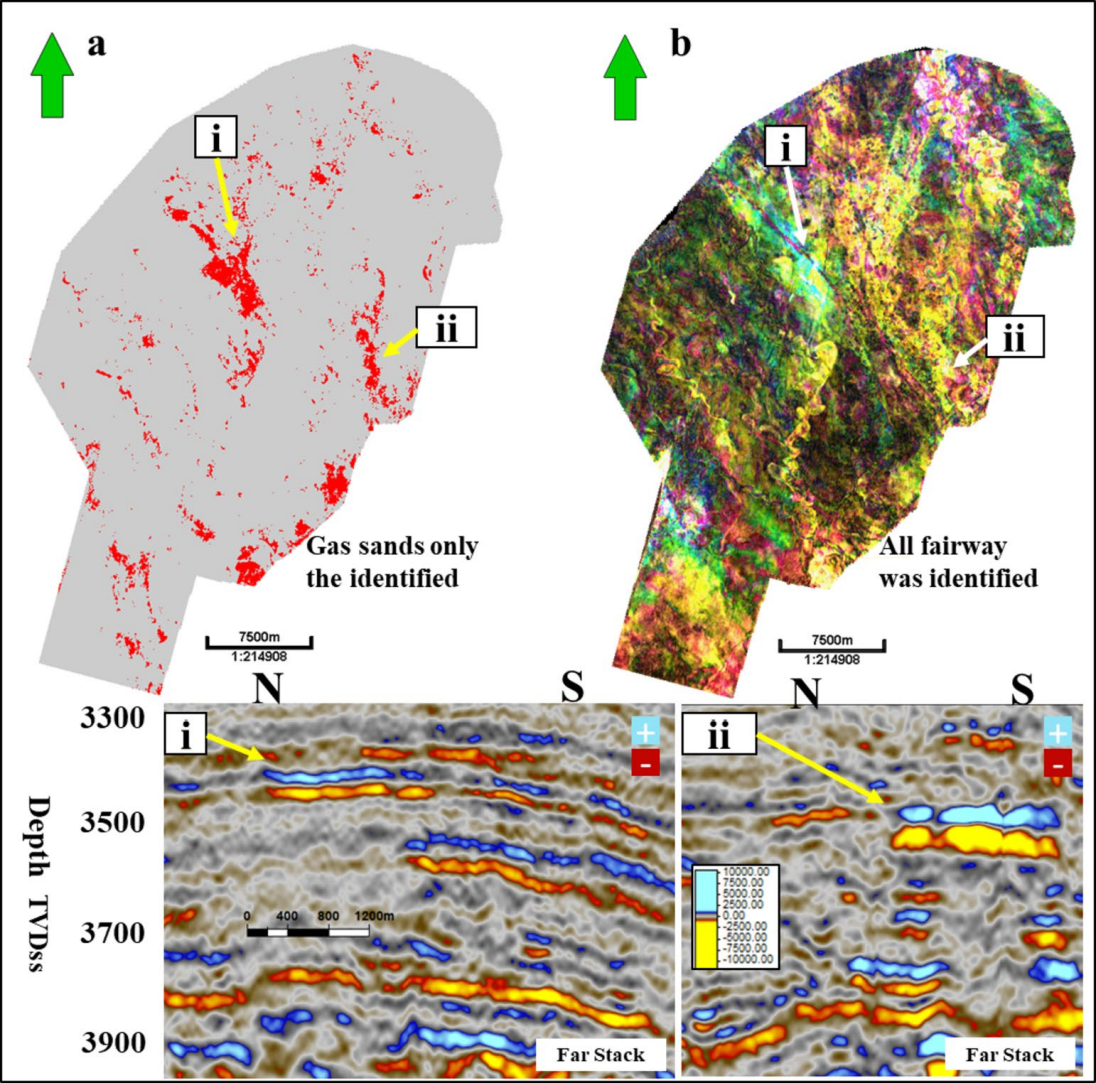

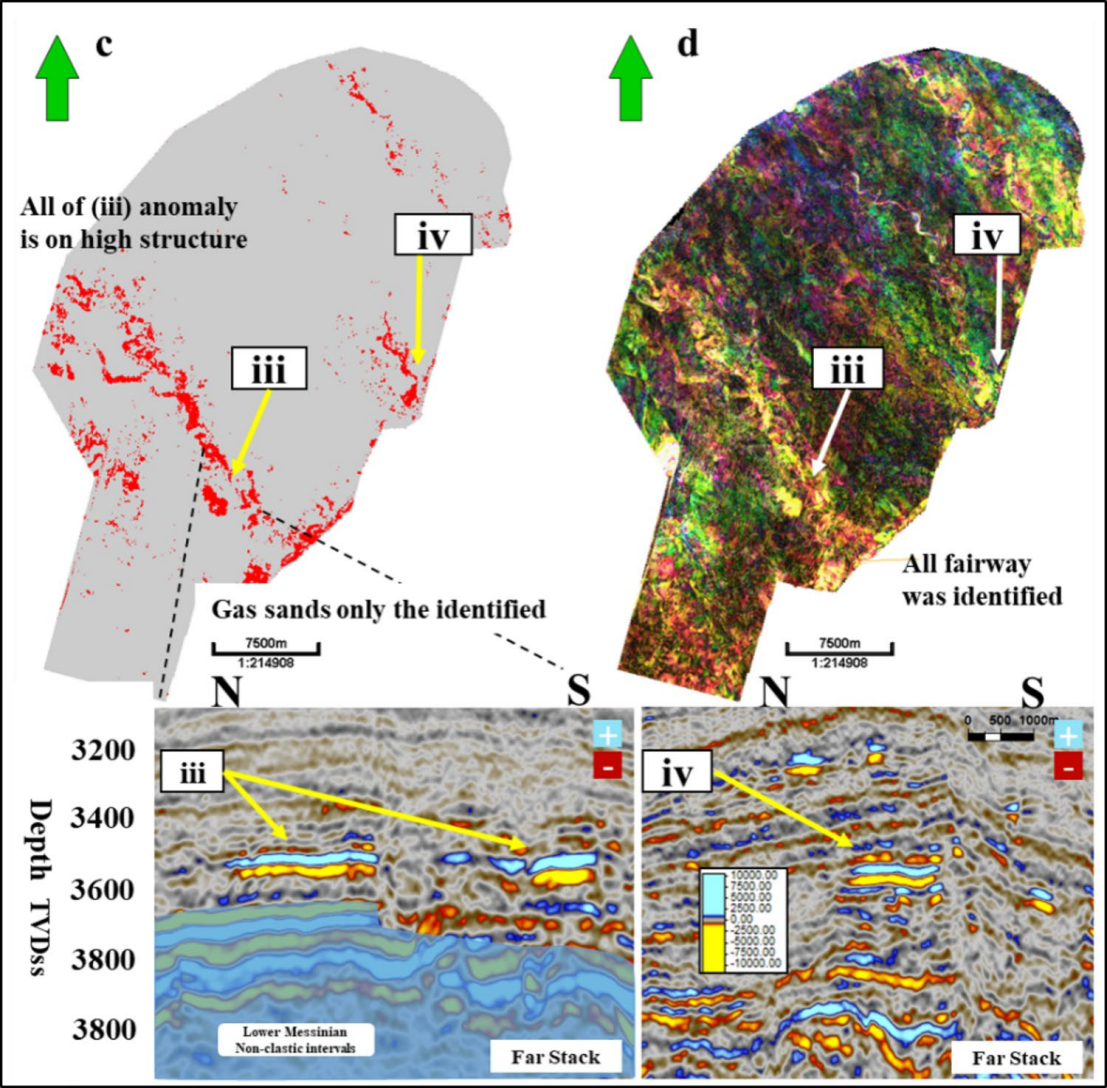
(**a**) 2D slice for gas sand cube with (i) and (ii) other examples on low seismic amplitude gas sand AVO class-II not identified before; (**b**) shows the same 2D slice on specteral decomposition and reveals the channel fairways for both gas sand and water sand, but gas sand only shows on gas sand 2D slice. (**c**) 2D slice for gas sand cube with (iii) and (iv) other example on low seismic amplitude gas sand AVO class-II not identified before, (**d**) shows the same 2D slice on specteral decomposition and reveals the channels fairways both gas sand and water sand part but gas sand only shows on gas sand 2D slice. Created in Rashpetco Egypt using Petrel 2023 sofware, www.slb.com.

Figure 16 shows a 2D horizon slice of the spectral decomposition for the Simian Pliocene field, as well as a 2D horizon slice of a gas sand cube for the Simian field. The 2D slices demonstrate the gas sand cube’s exceptional resolution specifically for the gas sand region. Figure 17 shows a 2D cross-section of the gas sand cube. It highlights four instances of low seismic amplitude gas sand AVO class II that were previously unidentified. Additionally, the figure presents the same two-dimensional slice on spectral decomposition. The spectral decomposition analysis identifies the pathways for both gas sand and water sand components. However, only the gas sand component is visible in the gas sand 2D slice.

When there is minimal variation in impedance or seismic amplitude among gas sand, water sand, and shale, traditional seismic interpretation methods may struggle to distinguish between these layers effectively. In such scenarios, machine learning (ML) techniques offer a significant advantage, as they can detect subtle patterns in the data that differentiate between the normal geological background and key features like the reservoir and aquifer. ML models can leverage multiple seismic attributes simultaneously, improving their sensitivity to variations that might be indistinguishable by conventional analysis alone.

**Fig. 18:**
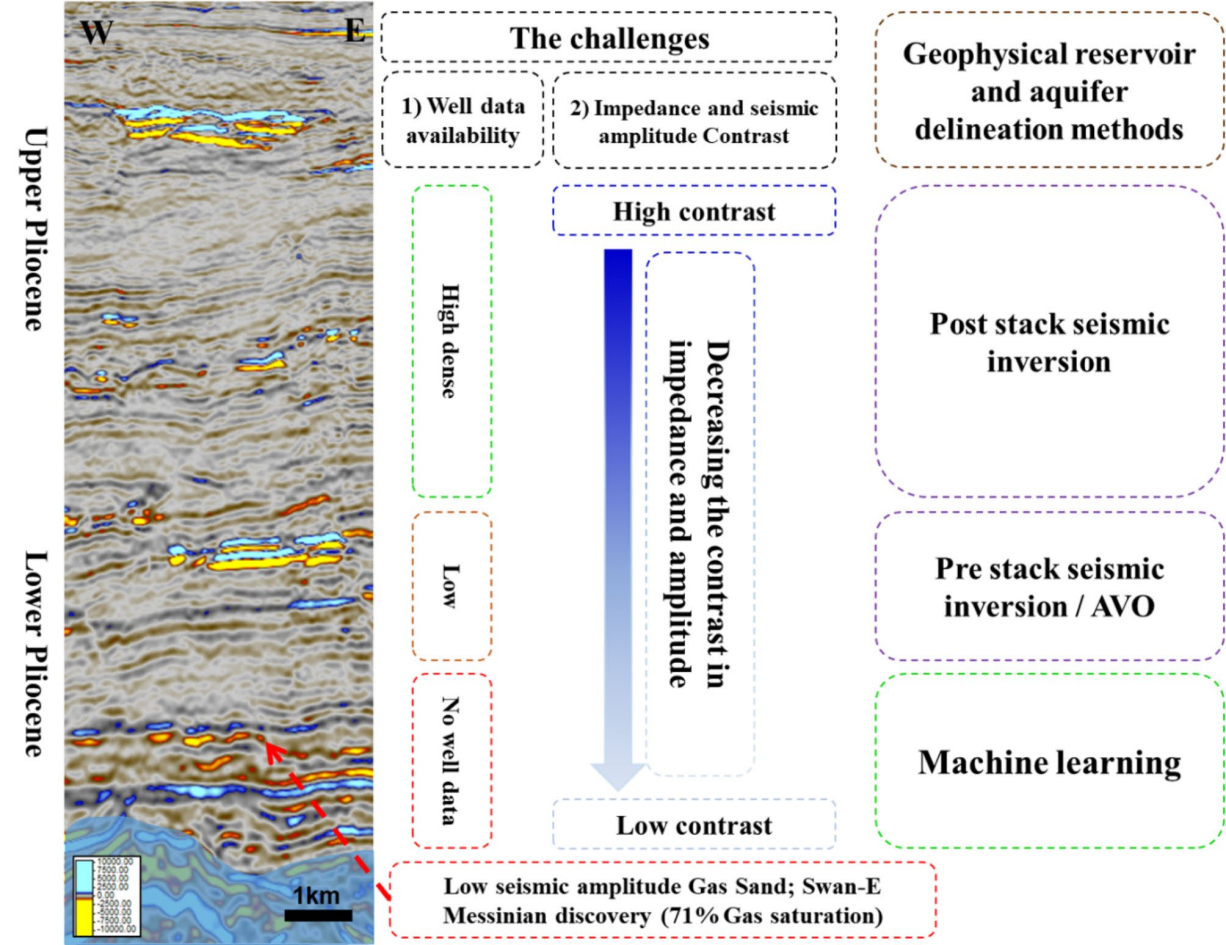
shows the challenges in lower Pliocene and the suitable geophysical tool for reservoirs and aquifer delineations.

Additionally, in the absence of well data, conventional approaches such as pre-stack seismic inversion and AVO (amplitude versus offset) modeling face limitations in accurately delineating reservoirs and aquifers, as these methods typically rely on well information for calibration and validation. ML, on the other hand, can extrapolate patterns learned from available seismic data, allowing for more effective prediction and mapping of reservoir and aquifer boundaries even when well data is sparse or unavailable. Consequently, ML enhances subsurface characterization, improving the reliability of prospect identification in complex geological settings. Figure 18 shows these challenges and the suitable geophysical approaches for reservoir and aquifer delineation.

A comparative analysis of the outputs from the machine learning, amplitude versus offset, and seismic inversion workflows demonstrates notable advantages in the machine learning methodology for prospect identification. The classification cube produced by machine learning exhibits enhanced resolution and precision in identifying hydrocarbon-bearing zones relative to conventional techniques. AVO analysis examines variations in amplitude with offset to infer fluid content, while seismic inversion reveals changes in impedance that indicate lithological alterations. In contrast, the machine learning workflow integrates various seismic attributes and employs pattern recognition to create a more coherent and comprehensible subsurface model. The machine learning approach can more precisely delineate delicate stratigraphic traps and intricate reservoir geometries due to the impedance of the water sand being similar to that of the shale background. This reduces the ambiguity in prospect and aquifer delineation. This integrated approach facilitates the identification of prospective exploration zones, as evidenced by more distinct border delineations and enhanced reliability metrics in the machine learning classification results. This enhances the precision and capacity for decision-making in geophysical interpretation^18–24^. Table 2 shows a detailed comparison between AVO analysis, seismic inversion, and neural networks.

**Table 2:** Comparison among AVO (amplitude variation with offset), weismic inversion, and neural networks for prospect and aquifer delineation.

Aspect	AVO	Seismic Inversion	Neural Networks
**Primary Objective**	Detects changes in amplitude with offset to infer fluid types and lithology contrasts.	Converts seismic data into impedance models for detailed subsurface property characterization.	Uses multiple seismic attributes to learn patterns for automated identification of lithology and fluid types.
**Data Requirements**	Requires pre-stack seismic data and well information for reliable calibration.	It requires post-stack seismic data, with pre-stack used for elastic inversion. Well control is essential for calibration.	Requires dataset with labeled (seismic attribute data) for training and validation.
**Prospect Delineation**	Effective for detecting gas and hydrocarbon indicators based on amplitude responses related to fluid content.	Provides continuous impedance models that reveal lithology and fluid-related properties, aiding in detailed reservoir characterization.	Offers automated prospect delineation, integrating multiple attributes to identify subtle features that may be overlooked by traditional methods.
**Aquifer Maping**	Good in high intercept contrast between water sand and shale and limit in case of low intercept contrast	Good in high impedance contrast between water sand and shale and limit in case of low impedance contrast	Good aquifer mapping in cases of high or low impedance contrast
**Resolution**	Good lateral resolution but can be limited by far-offset noise and requires calibration for depth accuracy.	High vertical resolution that captures thin layers and stratigraphic boundaries when well calibrated.	Resolution depends on input data quality but can learn complex patterns to enhance resolution and interpretability.
**Output Data Type**	Produces AVO attributes, like intercept and gradient, used to infer fluid and lithological contrasts.	Outputs impedance volumes (acoustic or elastic), which provide continuous property estimates.	Produces classification or regression results (e.g., classification cubes, probability maps), indicating likely prospects.
**Interpretation Complexity**	Requires expertise in AVO interpretation to distinguish fluid and lithology effects, prone to false positives from tuning effects.	Less ambiguity than AVO in some cases, but may still need calibration for accurate lithology and fluid predictions.	Automated; reduces subjective interpretation but requires expertise to tune and validate model outputs.
**Limitations**	Sensitive to noise and tuning effects, it may produce misleading results without proper angle coverage and calibration.	Computationally intensive and sensitive to model assumptions; effectiveness depends on well calibration.	Requires a large, representative training dataset; is prone to overfitting and may struggle with generalization to new areas without extensive data.
**Strength in Prospecting**	Particularly useful for hydrocarbon detection, especially gas, in high-porosity reservoirs.	Effective in delineating lithology, porosity, and subtle stratigraphic features, ideal for complex reservoirs.	Excel excels at integrating multiple data sources, revealing complex patterns, and automating prospect and aquifer identification, which can reduce uncertainty in exploration.

This research represents a significant contribution to the field of hydrocarbon exploration by developing a novel workflow for detecting low seismic amplitude gas fields, which are often difficult to identify using conventional methods. One of the key innovations is the integration of seismic spectral decomposition, AVO analysis, and machine learning techniques, providing a more advanced approach compared to traditional seismic analysis.

Existing studies have typically focused on high-amplitude seismic anomalies or relied on standard seismic interpretation techniques, which often struggle to detect subtle gas fields with low seismic amplitude responses. This study, on the other hand, uses spectral decomposition to see small differences, draw complicated stratigraphic features like low seismic amplitude incised channels, and find the aquifer in the fairway. This lowers the risk of finding low seismic amplitude gas reservoirs and their hydrocarbon accumulations. This allows for more precise boundary definition of low seismic amplitude gas sands, a crucial improvement for accurately identifying hydrocarbon reservoirs.

Additionally, the application of the AVO (amplitude versus offset) classification further distinguishes this work from previous research. By validating gas sand with a water leg using spectral decomposition, the workflow provides enhanced accuracy in lithology and fluid classification, which is often a challenge in complex geological settings within the deeper section of low seismic amplitude gas reservoirs.

The integration of machine learning algorithms marks another key contribution. By classifying seismic facies and isolating low seismic amplitude anomalies from seismic background aquifers from seismic background non-anomalous data, the study enhances the predictive capability of exploration projects. This approach reduces the uncertainty and risk of drilling unsuccessful wells, a critical issue in hydrocarbon exploration.

Finally, the validation of this workflow using a blind section and the application of machine learning to generate probability cubes for facies domains show that this method can generalize beyond the specific study area. Thus, the research not only advances current methodologies but also holds significant potential for application in other deep-water exploration settings, providing a more reliable way to assess low seismic amplitude hydrocarbon prospects.

The success rate in drilling is a critical benchmark for assessing the effectiveness of machine learning (Neural Network) in prospect delineation, as it directly measures the model’s ability to accurately identify productive drilling locations. This metric compares the percentage of wells that produce hydrocarbons when drilled based on machine learning predictions versus those selected using traditional methods, such as AVO and seismic inversion. A higher drilling success rate with machine learning-guided predictions suggests that the model is better at identifying subsurface features indicative of hydrocarbons, leading to fewer dry wells and improved exploration efficiency. By analyzing success rates, operators gain insights into the machine learning model’s practical reliability and economic impact. Higher drilling success rates reduce exploration costs by lowering the frequency of unproductive wells and increasing the likelihood of tapping into viable reservoirs. Consequently, an improved drilling success rate not only validates the machine learning approach but also enhances the decision-making process, making it a key indicator of the value that machine learning brings to exploration efforts. For instance, the AVO analysis and seismic inversion failed to mitigate the risk associated with the high-water saturation prospect in the Sinbad field within the same research region^‎9^.

The limitations of this study are primarily related to the complexity of detecting low seismic amplitude gas fields and the inherent challenges posed by seismic data quality. The workflow relies heavily on spectral decomposition and AVO analysis, which can be sensitive to data resolution and signal-to-noise ratios. Low-quality seismic data may result in less precise identification of subtle anomalies, particularly in areas where the gas sand exhibits low contrast with the surrounding water sand or shale.

Another limitation is the dependency on machine learning algorithms, which, while powerful, are only as good as the input data used to train them. If the training data set lacks diversity or does not fully capture the range of geological conditions present in the study area, the model may struggle with generalization when applied to different seismic volumes. There is also the risk of overfitting the model, which could compromise its performance when applied to new, unseen data.

The accuracy of AVO classification is another challenge, especially when distinguishing between gas sand, water sand, and shale. In complex geological structures such as faulted zones or areas with rotated fault blocks, AVO responses can be ambiguous, leading to potential misclassification. Furthermore, while spectral decomposition is effective at identifying certain geological features like incised channels, it may not detect more subtle stratigraphic traps or features that do not present strong frequency contrasts.

The study’s reliance on broadband seismic acquisition aims to enhance low-frequency content for deeper target detection. However, the dominant frequency of 18 Hz, while useful, may limit the resolution of deeper, thinner reservoirs, potentially affecting the accuracy of the results. Additionally, the workflow developed for the West Delta Deep Marine (WDDM) concession might require adaptations when applied to other geological settings or exploration areas.

Finally, while the workflow was validated using a blind section that excluded a known shallow gas field, there remains a level of uncertainty in the interpretation of exploration prospects. False positives or negatives could have significant economic implications, particularly when it comes to the drilling of wells based on this analysis.

## Conclusion

In this study, we developed a comprehensive workflow to detect and characterize low seismic amplitude gas fields in the West Delta Deep Marine (WDDM) concession offshore Egypt. The workflow utilized advanced seismic spectral decomposition techniques and machine learning algorithms to identify subtle anomalies associated with low seismic amplitude gas sand, water sand, and shale backgrounds. Spectral decomposition was employed to detect incised channels and define the fairway of the prospects. This technique allowed for the visualization of subtle features that were not easily discernible on standard seismic data as having the same background amplitude. AVO analysis was then used to classify the anomalies and determine their lithology and fluid properties.

Machine learning algorithms were applied to the seismic data to identify and differentiate low seismic amplitude gas sand anomalies from water sand and shale backgrounds. The neural network was trained by using known examples of low seismic amplitude gas sand, water sand, and shale and then applied to the seismic volume to generate probability cubes for each target feature.

Validation of the workflow was performed by testing the results on a blind section, which excluded a known shallow gas field. The results showed good agreement with the known geology, confirming the effectiveness of the workflow in identifying potential hydrocarbon reservoirs.

Overall, the study demonstrates the value of integrating advanced seismic analysis techniques and machine learning algorithms in the exploration and delineation of low seismic amplitude hydrocarbon reservoirs in complex deep-water settings. The workflow developed in this study provides a systematic approach to reduce exploration risk and improve the accuracy of reservoir characterization in similar geological settings.

## Data Availability

The data that support the findings of this study are available on request from the corresponding author.
